# The safety and efficacy of direct oral anticoagulants among chronic kidney disease patients on dialysis with non-valvular atrial fibrillation: a meta-analysis

**DOI:** 10.3389/fcvm.2023.1261183

**Published:** 2023-09-18

**Authors:** Jerahmeel Aleson L. Mapili, Lloyd Christopher S. Lim, Bianca M. Velando, Jaime Alfonso M. Aherrera

**Affiliations:** Division of Cardiovascular Medicine, Department of Medicine, University of the Philippines - Philippine General Hospital, Manila, Philippines

**Keywords:** atrial fibrillation, direct oral anticoagulant, chronic kidney disease, dialysis, end-stage renal disease (ESRD)

## Abstract

**Background:**

Individuals with chronic kidney disease (CKD) on dialysis are at an increased risk of stroke and embolic events especially in the presence of atrial fibrillation (AF). Vitamin K antagonists (VKA), including warfarin, have been used for decades for anticoagulation among CKD patients on dialysis with AF but recent evidence has shown increased bleeding. Direct oral anticoagulants (DOAC) have been emerging as an alternative to VKA which, based on several observational cohort studies, are at least as efficacious and safe as VKA. This meta-analysis looked into the safety and efficacy of DOACs compared to VKA among CKD patients on dialysis with non-valvular AF.

**Methodology:**

This study used a random-effects meta-analysis using RevMan 5.4. PubMed, EMBASE, Cochrane Central Register of Controlled Trials, Cochrane Database of Systematic Reviews, and ClinicalTrials.gov were searched from their dates of inception to June 2023. The risk of bias was assessed using Cochrane RoB2 and the certainty of evidence was assessed using GRADE.

**Results:**

This meta-analysis showed that DOACs when compared to VKA have no significant difference in terms of risk for major bleeding (RR = 0.81, 95% CI 0.46–1.43), ischemic stroke (RR = 0.5, 95% CI 0.19–1.35), and cardiovascular death (RR = 1.34, 95% CI 0.69–2.60).

**Discussion:**

This meta-analysis adds to the growing body of evidence supporting that the use of DOACs has similar efficacy and safety outcomes in CKD patients on dialysis with non-valvular AF patients compared to VKA. The findings need to be replicated in larger and more adequately powered clinical trials in order to ascertain its level of evidence.

## Introduction

Patients with end-stage kidney disease (ESRD) are at higher risk of stroke or systemic thromboembolism (1). Among individuals with chronic kidney disease (CKD), atrial fibrillation (AF) is associated with a six-fold increase in stroke risk and is associated with accelerated progression of CKD which leads to increased mortality (2, 3). AF remains as a common indication for anticoagulation in patients with chronic kidney disease (CKD) (4).

Warfarin, a vitamin K antagonist (VKA), has been used for anticoagulation in ESRD for decades but recent evidence has shown that it has an increased bleeding risk compared to those without anticoagulation in this particular population (5, 6). Direct oral anticoagulants (DOAC) are emerging as alternatives to warfarin among patients requiring anticoagulation for AF as several trials have shown superior efficacy and safety compared to warfarin among patients with CKD who are not on dialysis (7). However, the efficacy and safety of DOAC among patients with CKD on dialysis remains understudied. A meta-analysis in 2022 on one randomized trial and five retrospective cohorts showed that DOACs had similar efficacy and safety among AF patients on dialysis compared to VKA (7). As a corollary, several recommendations discourage the use of DOAC among patients on maintenance dialysis. Hence there are several ambiguities regarding its use in CKD.

Two recent trials, the AXADIA-AF NET 8 and RENAL-AF, have shown that apixaban and VKA had similar efficacy and safety profiles while the VALKYRIE study showed that a reduced dose of rivaroxaban significantly decreased the composite outcome cardiovascular events with less major bleeding complications compared with VKA (8–10). These three trials however were underpowered and were inconclusive with regard to the efficacy and safety of direct oral anticoagulants among patients on dialysis. This meta-analysis looked into the safety and efficacy of DOACs among CKD patients on dialysis with non-valvular AF.

## Objectives

This meta-analysis assessed the following:

(1) Safety of direct oral anticoagulants in terms of major bleeding among chronic kidney disease patients on dialysis with non-valvular atrial fibrillation
(2) Efficacy of direct oral anticoagulants in terms reduction in ischemic stroke and cardiovascular death among chronic kidney disease patients on dialysis with non-valvular atrial fibrillation

## Methodology

### Study eligibility criteria

#### Inclusion criteria

The meta-analysis included the studies which met the following criteria: (1) Randomized controlled trial as its study design, (2) Intervention includes direct oral anticoagulant (regardless of mechanism of action), (3) Population are adults with atrial fibrillation on anticoagulation for stroke prevention with chronic kidney disease on maintenance dialysis, (4) Efficacy outcome reported includes ischemic stroke prevention, and (5) safety outcome reported should include major bleeding using any standard criteria.

#### Exclusion criteria

The meta-analysis excluded the studies if they met any of the following: (1) Study design is not a randomized controlled trial, (2) not in the English language or published in recognized international journals, (3) population includes patients with non-dialytic chronic kidney disease, (4) outcomes do not included those specified as key objective variables.

### Literature search strategy

The PRISMA (Preferred Reporting Items for Systematic Reviews and Meta-Analyses) guidelines was used for the conduct of the literature search (11). An independent electronic search was performed by three investigators (JAM, LCL, and BMV) using PubMed, EMBASE, Cochrane databases, and ClinicalTrials.gov from their dates of inception to June 2023. The following search terms were used: “direct oral anticoagulant”, “novel oral anticoagulant”, “chronic kidney disease”, “dialysis”, “end-stage renal disease” as free text and/or as MeSH terms. Additionally, a maximally sensitive search was sought by using the search terms for randomized and clinical trials (12, 13). Furthermore, an independent review of reference lists was done to identify additional potentially relevant studies.

An independent review for duplicates were done by the three investigators (JAM, LCL, and BMV) after the initial electronic search to complete the identification phase. A review of abstracts alone was done for the screening phase. Studies which did not meet the inclusion criteria were outright ineligible for review. After screening the abstracts, a full-text review of eligible studies was done. Hence, studies which met the inclusion were included in the final meta-analysis.

In the event of discrepancy in the independent literature search, a consensus was made via a discussion and consultation with the fourth investigator (JMA).

### Data extraction and critical appraisal

An independent review of each article by three investigators (JAM, LCL, and BMV) was done for data extraction of the variables for analysis. The study design, intervention, control, and outcomes were extracted from the full text articles including their appendices and supplementary files (if available).

The Revised Cochrane Risk-of-Bias Tool for Randomized Trials (RoB2) tool was used to assess the risk of bias in each study. This was independently assessed by three investigators (JAM, LCL, and BMV) (14). The risk of bias table is color coded as follows: green for low risk, orange for unknown risk, and red for high risk; an explanation for the risk of bias will also be provided in the tabulation.

Overall quality of evidence for each outcome were independently assessed by three investigators (JAM, LCL, and BMV) based on the Grades of Recommendation, Assessment, Development and Evaluation (GRADE) Working Group system (15).

### Data synthesis and statistical analysis

Cochrane Review Manager version 5.4 was used for the data analysis (16). The relative risk (RR) was used as a summary statistic for dichotomous outcomes and was represented in the Forest plots with the 95% confidence interval.

For the overall summary statistic, the composite relative risk and 95% confidence interval is presented and is represented by the middle and width of the diamond, respectively. To assess heterogeneity, we used the *I*^2^ statistic with values greater than 50% considered as substantial heterogeneity. In the event of substantial heterogeneity, a subgroup analysis was done.

## Results

### Literature search

**Figure 1:**
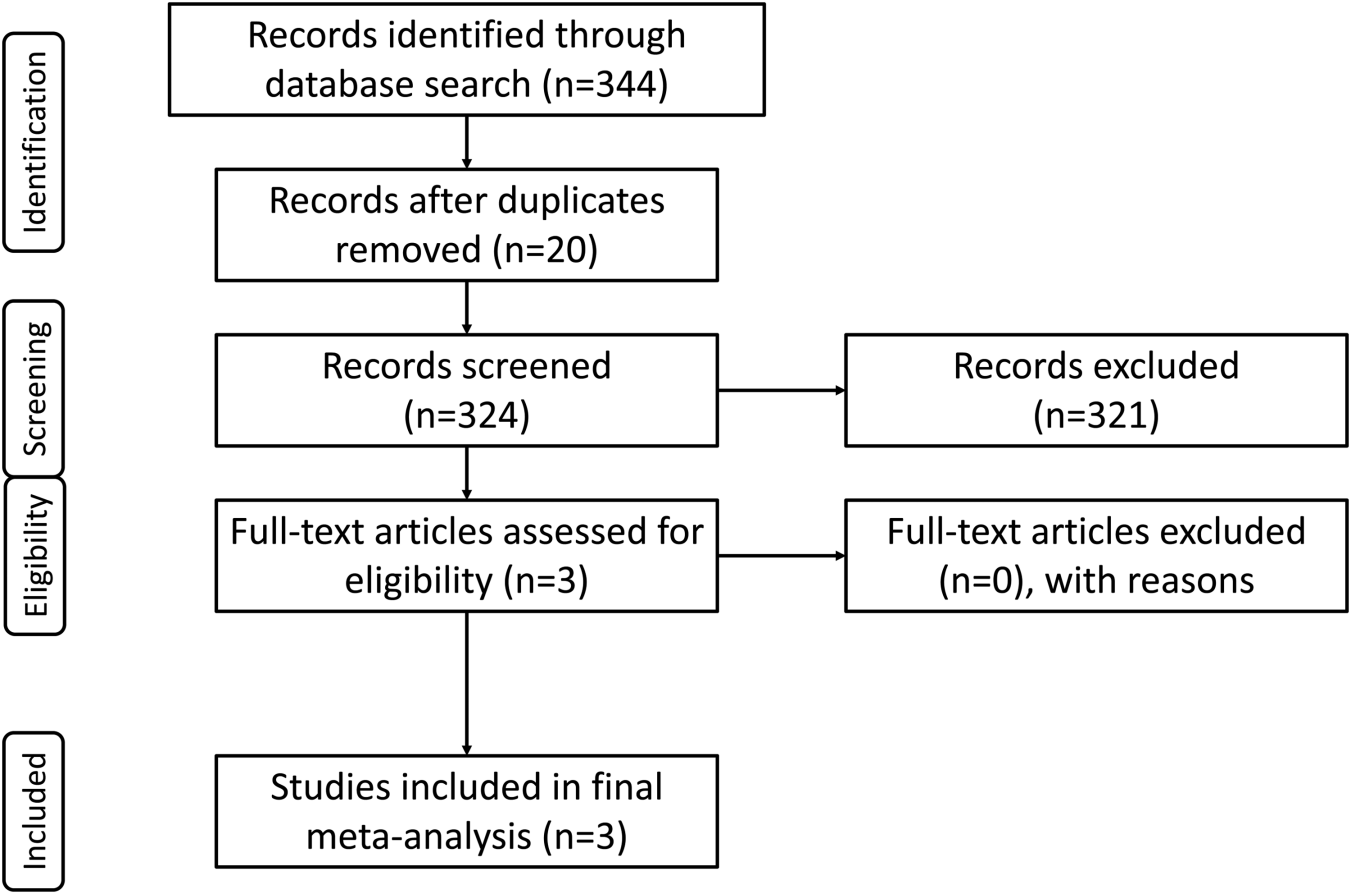
PRISMA summary of literature search.

The consensus electronic literature search yielded a total of 344 studies. After the removal of 20 duplications, the remaining studies underwent primary screening of their abstracts. Following removal of 321 studies upon screening, three (3) studies underwent full-text review, and these three (3) were included in the final meta-analysis based on inclusion and exclusion criteria (Figure 1).

### Description of selected studies

The three selected trials (Table 1) were similar in terms of their control arm (VKA to target INR 2-3) and pre-specified outcomes of interest but they differed in their respective intervention arms. The VALKYRIE and AXADIA-AFNET 8 trials utilized a reduced dose DOAC while the RENAL-AF trial utilized the standard dose DOAC with weight and age adjustments as needed.

**Table 1:** Comparison of selected trials.

Trial	Intervention	Control	Efficacy outcomes	Safety outcomes
VALKYRIE (2021)	Rivaroxaban 10 mg OD	VKA, target INR 2-3	fatal cardiovascular disease and nonfatal stroke, cardiac events, and other vascular events	ISTH major bleeding
RENAL-AF (2022)	Apixaban 5 mg BID or 2.5 mg BID (if age >80 or weight <60 kg)	VKA, target INR 2-3	stroke, systemic embolization, cardiovascular death	ISTH major bleeding
AXADIA- AFNET 8 (2022)	Apixaban 2.5 mg BID	VKA, target INR 2-3	myocardial infarction, ischemic stroke, all-cause and cardiovascular death, and venous thromboembolism	ISTH major bleeding

### Baseline characteristics

The baseline characteristics (Table 2) of each of the studies were similar between treatment and control groups individually but have slight differences when compared to each other. The mean age for each trial varied by around 5 years from each other but nonetheless the mean ranged from 68 to 80 years old. There was no difference in terms of baseline average CHADSVASC and HASBLED scores between and among the trials.

**Table 2:** Baseline characteristics of selected trials.

	VALKYRIE (2021)		RENAL-AF (2022)		AXADIA- AFNET 8 (2022)	
	VKA	DOAC	VKA	DOAC	VKA	DOAC
Age (year)	80.3	79.9	68	69	74.8	74.7
Male sex (%)	56.8	76.1	69.4	58.5	75.5	64.6
CHA_2_DSV_2_ASc score	4.8	4.7	4.0	4.0	4.54	4.5
HAS-BLED score	4.7	4.6	No mention	No mention	4.15	4.25

### Safety outcomes

#### Major bleeding

**Figure 2:**
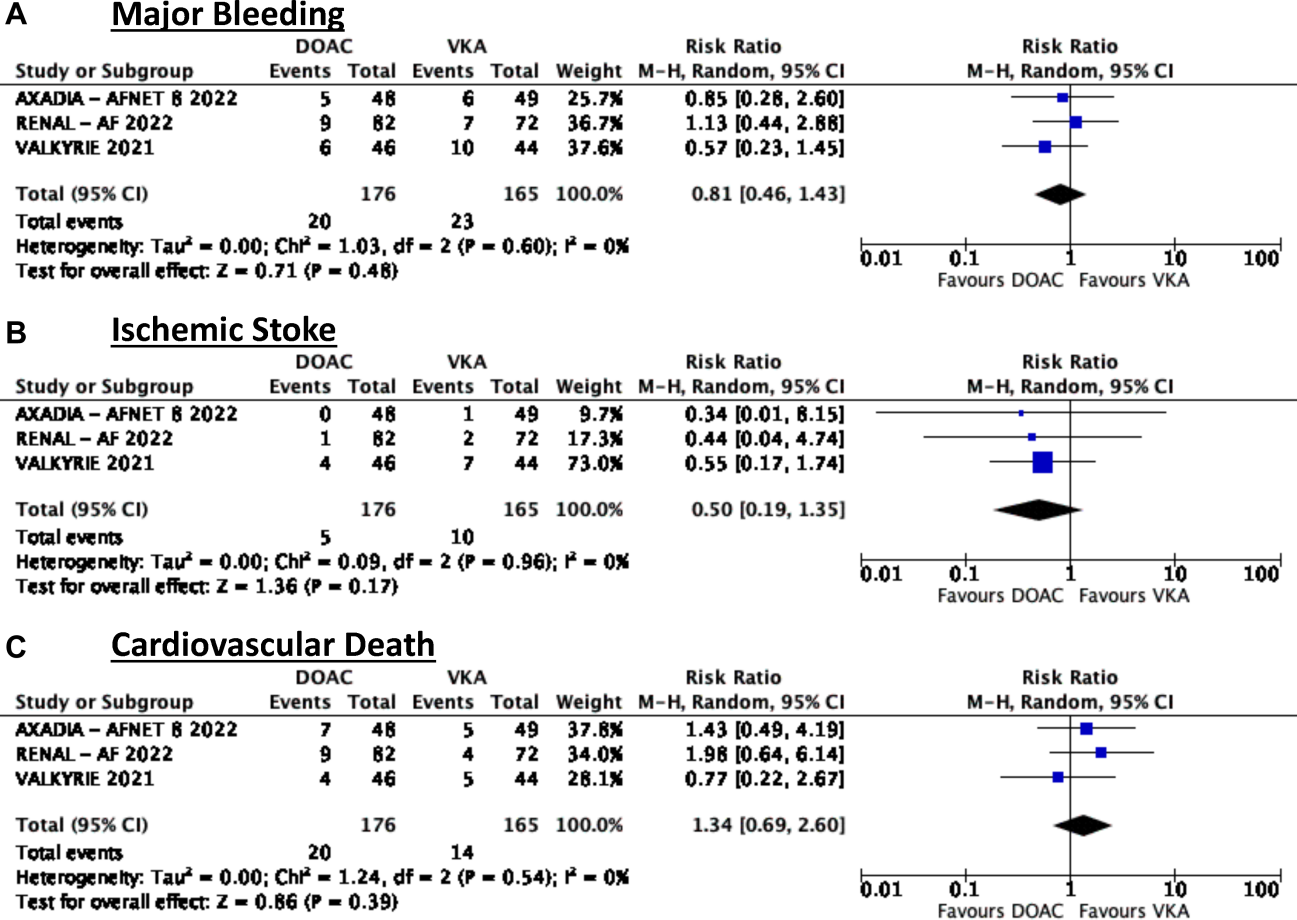
Forest plots summarizing the effects of intervention.

In terms of safety, this meta-analysis showed that among patients on dialysis with atrial fibrillation there was no significant difference in terms of major bleeding (RR = 0.81, 95% CI 0.46–1.43). There was no noted heterogeneity (Figure 2A).

### Efficacy outcomes

#### Ischemic stroke

In terms of efficacy, there was also no noted significant difference in terms of reduction in ischemic stroke (RR = 0.5, 95% CI 0.19–1.35) (Figure 2B).

#### Cardiovascular death

Similarly, it was noted that there was also no significant difference between the two treatment groups (RR = 1.34, 95% CI 0.69–2.60) (Figure 2C).

### Evaluation of studies

The risk of bias of the studies assessed using the Revised Cochrane Risk-of-Bias 2 tool for Randomized Trials (RoB2) showed that there was no risk of bias among all the studies included in this meta-analysis (Table 3). Hence overall, this meta-analysis had low risk of bias.

**Table 3:** Risk of bias table.

Author/trial (year)	Selection bias				Performance bias		Detection bias		Attrition bias		Reporting bias		Other biases	
	Random sequence generation		Allocation concealment		Blinding of participants		Blinding of outcome assessment		Loss to follow-up		Selective outcome reporting?		Funding?	
VALKYRIE (2021)														
RENAL-AF (2022)														
AXADIA-AFNET8 (2022)														

Using the GRADE approach to assess quality of evidence (Table 4), major bleeding and ischemic stroke had moderate quality of evidence based mainly on the insignificant treatment effect. The quality of evidence on cardiovascular death was deemed low due to insignificant results and the heterogeneity in the results between apixaban and rivaroxaban.

**Table 4:** Summary of findings.

Outcomes	Anticipated absolute effects* (95% CI)		Relative effect (95% CI)	№ of participants (studies)	Certainty of the evidence (GRADE)
	Risk with VKA	Risk with DOAC			
Major bleeding	139 per 1,000	**113 per 1,000** (64–199)	**RR 0.81** (0.46–1.43)	341 (3 RCTs)	⊕⊕⊕⃝ Moderate^a^^,^^b^
Ischemic stroke	61 per 1,000	**30 per 1,000** (12–82)	**RR 0.50** (0.19–1.35)	341 (3 RCTs)	⊕⊕⊕⃝ Moderate^b^
Cardiovascular death	85 per 1,000	**114 per 1,000** (59–221)	**RR 1.34** (0.69–2.60)	341 (3 RCTs)	⊕⊕⃝⃝ Low^b^^,^^c^

^*^ Means estimate from relative effect per 1,000.

^a^ RENAL-AF Trial showed a trend towards increased bleeding.

^b^ Statistically not significant effect.

^c^ Trials on apixaban showed a trend towards increased cardiovascular death.

## Discussion

There is a huge debate regarding the anticoagulation strategy among patients with atrial fibrillation on chronic dialysis. There is a lack of consensus between the international guidelines (KDIGO 2012, ESC 2020, AHA/HRS 2019, CCS 2020, CHEST 2018), and most would either recommend a VKA or no anticoagulation while some guidelines state that standard or reduced dose apixaban may be used with caution (17) (Table 5). In general, NOACs are NOT recommended among patients with advanced CKD (CrCl < 15 ml/min), more so those on hemodialysis. In 2018, apixaban (at 5 mg BID) use has been approved by US FDA for AF in ESRD but has only been supported by pharmacokinetic studies. Hence this clinical scenario had to be elucidated even further. Based on the available evidence, this is the first meta-analysis which investigates the efficacy and safety profiles of DOACs vs. VKA in patients with AF undergoing dialysis which only analyzed randomized controlled trials.

**Table 5:** Comparison of current guidelines.

Guideline (year)	Recommendations
KDIGO (2012)	Lower doses of warfarin with close monitoring when eGFR < 30 ml/min. Routine anticoagulation in patients with CKD stage 5 on dialysis is not recommended for primary prevention of stroke.
ESC (2020)	None of the NOACs have been approved in Europe for patients with CrCl < 15 ml/min or on dialysis.
CCS (2020)	Warfarin is recommended with eGFR 15–30 ml/min and not on dialysis, but patients with AF receiving dialysis should not be prescribed oral anticoagulation or aspirin for stroke prevention.
CHEST (2018)	In ESRD (CrCl < 15 mlmin or dialysis-dependent), NOACs should generally not be used, but well-managed VKA with time-in-therapeutic range (TTR) >65%–70% (ungraded consensus-based statement) and individualized decision-making applies.
ACC/AHA/HRS (2019)	Warfarin and apixaban may be used without dose restrictions when CrCl < 15 ml/min and regardless of the need for RRT. However other NOACs to be avoided in ESRD patients and on RRT.

This meta-analysis showed that direct oral anticoagulants have no significant difference in terms of risk for major bleeding, ischemic stroke, and cardiovascular death. As previously mentioned, the three randomized controlled trials were rather underpowered individually and the confidence interval of each of the studies were rather wide. It can be noted however that in our pooled analysis, with more power from the increase in the sample size to a total of 341, the confidence interval invariably narrows for each of the respective outcomes of interest. A recent meta-analysis done in 2022, which included the VALKYRIE trial and five other observational studies, showed that the use of DOAC, especially rivaroxaban or apixaban, showed at least similar efficacy and safety among AF patients on dialysis (7).

In terms of bleeding risk, the pooled analysis showed no significant difference but it should be noted that the AXADIA-AFNET 8 and VALKYRIE trials showed a trend towards less major bleeding while the RENAL-AF trial showed a trend towards increased risk of bleeding. This can be due to the differences in dosing used in the trials and the time in therapeutic range (TTR) achieved in each trial. It should be noted that the two trials which showed a trends towards less bleeding utilized a reduced dose DOAC with apixaban 2.5 mg BID and rivaroxaban 10 mg OD respectively while the RENAL-AF trial utilized a standard dose apixaban 5 mg BID. It should also be noted that the TTR for patients in the VKA arm was low at 44.3% in the RENAL-AF trial indicating that the estimate for bleeding events may be underestimated in the VKA arm. In contrast, the VALKYRIE Trial had a larger TTR of 48% at 6 months, was steady at 55%–69% from month 6 to 54, and increased to 87% at 54–60 months. Meanwhile, the AXADIA-AF NET 8 had a TTR of 50.7% in the VKA group. These differences in TTR in the VKA arm may be a key factor explaining why the RENAL-AF trial trended towards an increased risk of bleeding with DOAC use. Indeed, achieving an acceptable TTR is also a difficulty several trials face when comparing DOAC and VKA. Currently, there is an ongoing trial, the SAFE-D Trial, which seeks to compare standard dose apixaban vs. reduced dose apixaban vs. VKA among AF patients on dialysis with a potential to further elucidate on these dose discrepancies (18).

Our meta-analysis suggests that rivaroxaban has a lower risk of bleeding when compared to apixaban but, in a retrospective cohort study of the Danish nationwide cohort in 2020, it was shown that rivaroxaban is associated with a higher risk of bleeding compared to apixaban (19). In another retrospective study in 2018, the ARISTOPHANES Study, the rates of major bleeding are also higher in the rivaroxaban group compared to the apixaban group (20). However, these studies did not indicate the percentage of the population with CKD on dialysis and hence cannot be generalized in this population. Indeed further studies need to be done in order to ascertain the safety profiles of each of the DOACs especially when compared against each other and especially in the context of CKD on chronic dialysis.

In terms of ischemic stroke risk reduction, it can be noted that all three trials showed a trend towards reduction of events and when taken as a whole, the confidence interval narrows even further. In the previously mentioned meta-analysis done in 2022, it was shown that there was no significant risk reduction for ischemic stroke with DOAC compared to VKA but was leaning toward the side of benefit (7). Based on these, there are indeed signals that DOACs, particularly the factor Xa inhibitors, are particularly more efficacious than VKA in preventing ischemic stroke in dialysis patients with AF.

In a retrospective study in 2018, standard-dose apixaban (5 mg BID) was associated with significantly lower rates of ischemic stroke compared to either warfarin or low-dose apixaban (2.5 mg BID). In addition, apixaban, irrespective of the dose used, was associated with lower major bleeding rates than warfarin further supporting the fact that apixaban may well be a safe alternative among AF patients on dialysis (21).

The findings on cardiovascular death outcomes are inconsistent and based on the Forest plots it seems that a reduced dose rivaroxaban is associated with a decreased risk of cardiovascular death when compared to apixaban at either dose. Among the DOACs, it has been shown that a low dose rivaroxaban (5 mg daily) on top of antiplatelet therapy is associated with a reduction in cardiovascular events among those with atherosclerotic cardiovascular disease on antiplatelet therapy (22). In addition, the AFIRE trial has shown that rivaroxaban at reduced dose of 10 mg OD was non-inferior to rivaroxaban plus antiplatelet therapy among patients with AF and stable ischemic heart disease in terms of reduction of cardiovascular events and was likewise associated with a decreased risk of bleeding (23). These observations seen with rivaroxaban have not been replicated with the other DOACs and suggests that rivaroxaban may be the DOAC of choice in terms of cardiovascular risk reduction. These studies however failed to include patients with CKD on dialysis and hence cannot be totally applicable to this subgroup.

It should also be noted that randomized trials utilizing edoxaban and dabigatran were not retrieved from the literature search and currently there are no trials on patients on hemodialysis with AF using these two agents. It can be noted that all DOACs are eliminated by the kidneys in varying degrees wherein dabigatran is 80% renally excreted and edoxaban is 50% renally excreted rivaroxaban and apixaban are renally excreted at rates of 35% and 27% respectively based on pharmacokinetic studies. This may shed light as to why rivaroxaban and apixaban are the two main DOACs used in the retrieved trials in this meta-analysis and is also what is reflected in real-world clinical practice.

Based on the results of this meta-analysis and the current evidence from several observational cohort studies, there is still a large gap in our understanding of the use of DOACs among patients with AF on chronic dialysis. Our meta-analysis has its limitations mainly from the inclusion of only three studies and having a pooled population of 341. Nonetheless, the results of our pooled analysis show promising results which need to be replicated in larger and more adequately powered randomized clinical trials with population sizes approximating the pooled analysis of 350 or more in order to ascertain the level of evidence. However, due to the ambiguity in the evidence available at present, there is still hesitancy regarding the enrollment of patients into these types of trials and thus poses challenges to present and future investigators.

## Conclusion

In conclusion, our meta-analysis adds to the growing body of evidence regarding the use of DOACs in AF patients on chronic dialysis. As already highlighted in previous observational cohort studies and meta-analyses of these cohort studies, DOACs are at least as efficacious and safe among patients with CKD on dialysis needing anticoagulation for non-valvular AF. Being a pooled analysis of smaller trials, our meta-analysis likewise provides more power and higher level of certainty to their individual findings. To shed more light on the topic, we recommend that larger scale multi-center randomized clinical trials be pursued.

## Data Availability

The original contributions presented in the study are included in the article/Supplementary Material, further inquiries can be directed to the corresponding author.

## References

[1] Kumar S Lim E Covic A Verhamme P Gale CP Camm AJ Anticoagulation in concomitant chronic kidney disease and atrial fibrillation. J Am Coll Cardiol. (2019) 74:2204–15. 10.1016/j.jacc.2019.08.1031 31648714

[2] McBane RD. End-stage renal disease, nonvalvular atrial fibrillation, and the warfarin dilemma. Mayo Clin Proc. (2020) 95:1099–101. 10.1016/j.mayocp.2020.04.023 32498768

[3] Goel N Jain D Haddad DB Shanbhogue D. Anticoagulation in patients with end-stage renal disease and atrial fibrillation: confusion, concerns and consequences. J Stroke. (2020) 22:306–16. 10.5853/jos.2020.01886 33053946 PMC7568986

[4] Chokesuwattanaskul R Thongprayoon C Tanawuttiwat T Kaewput W Pachariyanon P Cheungpasitporn W. Safety and efficacy of apixaban vs. warfarin in patients with end-stage renal disease: meta-analysis. Pacing Clin Electrophysiol. (2018) 41:627–34. 10.1111/pace.13331 29577340

[5] van Zyl M Abdullah HM Noseworthy PA Siontis KC. Stroke prophylaxis in patients with atrial fibrillation and end-stage renal disease. J Clin Med. (2020) 9:123. 10.3390/jcm9010123 31906546 PMC7019832

[6] Lee M Saver JL Hong K Wu Y Huang W Rao NM Warfarin use and risk of stroke in patients with atrial fibrillation undergoing hemodialysis. Medicine (Baltimore). (2016) 95:e2741. 10.1097/MD.0000000000002741 26871818 PMC4753914

[7] Li W Zhou Y Chen S Zeng D Zhang H. Use of non-vitamin K antagonists oral anticoagulants in atrial fibrillation patients on dialysis. Front Cardiovasc Med. (2022) 9. 10.3389/fcvm.2022.1005742 PMC9513185 36176998

[8] Reinecke H Engelbertz C Bauersachs R Breithardt G Echterhoff HH Gerß J A randomized controlled trial comparing apixaban with the vitamin K antagonist phenprocoumon in patients on chronic hemodialysis: the AXADIA-AFNET 8 study. Circulation. (2023) 147(4):296–309. 10.1161/CIRCULATIONAHA.122.062779 36335915 PMC9875840

[9] Pokorney SD Chertow GM Al-Khalidi HR Gallup D Dignacco P Mussina K Apixaban for patients with atrial fibrillation on hemodialysis: a multicenter randomized controlled trial. Circulation. (2022) 146:1735–45. 10.1161/CIRCULATIONAHA.121.054990 36335914

[10] De Vriese AS Caluwé R Van Der Meersch H De Boeck K De Bacquer D. Safety and efficacy of vitamin K antagonists versus rivaroxaban in hemodialysis patients with atrial fibrillation: a multicenter randomized controlled trial. J Am Soc Nephrol. (2021) 32(6):1474–83. 10.1681/ASN.2020111566 33753537 PMC8259651

[11] Moher D Liberati A Tetzlaff J Altman DG Antes G Atkins D Preferred reporting items for systematic reviews and meta-analyses: the PRISMA statement. J Clin Epidemiol. (2009) 62(10):1006–12. 10.1016/j.jclinepi.2009.06.005 19631508

[12] Lefebvre C Manheimer E Glanville J. Chapter 6: searching for studies. In: Higgins J Green S, editors. Cochrane handbook for systematic reviews of interventions. Version 5.1.0 (updated march 2011). The Cochrane Collaboration (2011) Available at: www.cochrane-handbook.org

[13] Agoritsas T Merglen A Courvoisier DS Combescure C Garin N Perrier A Sensitivity and predictive value of 15 PubMed search strategies to answer clinical questions rated against full systematic reviews. J Med Internet Res. (2012) 14(3):e85. 10.2196/jmir.2021 22693047 PMC3414859

[14] Sterne JAC Savovi J Page MJ Elbers RG Blencowe NS Boutron I Rob 2: a revised tool for assessing risk of bias in randomised trials. Br Med J. (2019) 366:l4898. 10.1136/bmj.l4898 31462531

[15] Schünemann H Brożek J Guyatt G Oxman A. GRADE handbook for grading quality of evidence and strength of recommendations. (Updated October 2013).

[16] Cochrane Collaboration. Review manager (RevMan) [computer program]. Version 5.4. Copenhagen: The Nordic Cochrane Centre, The Cochrane Collaboration (2020).

[17] Ktenopoulos N Sagris M Theofilis P Lionaki S Rallidis LS. Direct oral anticoagulants in patients on chronic dialysis and concomitant atrial fibrillation: a common clinical impasse. Front Biosci (Schol Ed). (2022) 14(3):21. 10.31083/j.fbs1403021 36137976

[18] Harel Ziv. Strategies for the management of atrial fibrillation in patients receiving dialysis (SAFE-D). Ongoing study. Available at: https://classic.clinicaltrials.gov/ct2/show/NCT03987711

[19] Bonde AN Martinussen T Lee CJY Lip GYH Staerk L Bang CN Rivaroxaban versus apixaban for stroke prevention in atrial fibrillation: an instrumental variable analysis of a nationwide cohort. Circ Cardiovasc Qual Outcomes. (2020) 13:e006058. 10.1161/CIRCOUTCOMES.119.006058 32283966

[20] Lip GYH Keshishian A Li X Hamilton M Masseria C Gupta K Effectiveness and safety of oral anticoagulants among nonvalvular atrial fibrillation patients: the ARISTOPHANES study. Stroke. (2018) 49:2933–44. 10.1161/STROKEAHA.118.020232 30571400 PMC6257512

[21] Siontis KC Zhang X Eckard A Bhave N Schaubel DE He K Outcomes associated with apixaban use in patients with end-stage kidney disease and atrial fibrillation in the United States. Circulation. (2018) 138:1519–29. 10.1161/CIRCULATIONAHA.118.035418 29954737 PMC6202193

[22] Chen C Kan Y Shi Z Guo D Fu W Li Y Low dose rivaroxaban for atherosclerotic cardiovascular diseases: a systematic review and meta-analysis. Front Pharmacol. (2021) 11:608247. 10.3389/fphar.2020.608247 33732144 PMC7957832

[23] Yasuda S Kaikita K Akao M Ako J Matoba T Nakamura M Antithrombotic therapy for atrial fibrillation with stable coronary disease. N Engl J Med. (2019) 381:1103–13. 10.1056/NEJMoa1904143 31475793

